# Physical exercise and psychological resilience in the link between adverse childhood experiences and non-suicidal self-injury: chain mediation and network analysis in college students

**DOI:** 10.3389/fpsyt.2026.1889977

**Published:** 2026-08-12

**Authors:** Faxiang Fan, Hao Gou, Pan Liu, Luyao Xiang, Yi Huang

**Affiliations:** 1 College of Physical Education, Qiannan Normal University for Nationalities, Duyun, China; 2 Sports Institute of Chengdu University of Technology, Chengdu, China; 3 Zunyi Medical University, Zhuhai, China; 4 The First Affiliated Hospital of Zhengzhou University, Zhengzhou, China

**Keywords:** adverse childhood experiences, college students, network analysis, non-suicidal self-injury, physical exercise, psychological resilience

## Abstract

**Background:**

Non-suicidal self-injury is highly prevalent among college students, and adverse childhood experiences are an important risk factor. Physical exercise and psychological resilience may serve as protective factors in this association, while the item-level structure of NSSI behaviors requires further clarification.

**Methods:**

A cross-sectional design was adopted. A total of 2,626 college students were assessed using the Adverse Childhood Experiences Scale, the Physical Activity Rating Scale (PARS-3), the Psychological Resilience Scale, and the Non-Suicidal Self-Injury Behavior Assessment Questionnaire. Chain mediation analysis was conducted using SPSS 27.0 to examine theory-driven statistical indirect effects, and psychological network analysis was performed using R 4.3.2 to identify relatively central symptom nodes.

**Results:**

Adverse childhood experiences were significantly and positively associated with non-suicidal self-injury, and the PROCESS Model 6 analysis indicated a statistically significant indirect association through lower physical exercise and weaker psychological resilience. Further network analysis showed that “deliberately pinching oneself” had the highest expected influence and was involved in the relatively strongest edges in the NSSI symptom network.

**Conclusion:**

This study supported the statistical indirect roles of physical exercise and psychological resilience in the association between adverse childhood experiences and non-suicidal self-injury, and suggested that “pinching” was a noteworthy symptom in the NSSI behavioral network. The chain mediation and network analyses should be understood as complementary but parallel analyses, providing evidence at the total-score and symptom-item levels, respectively.

## 1 Introduction

Non-suicidal self-injury (NSSI) refers to the deliberate destruction of one’s own body tissue without suicidal intent and has become an important mental health concern among college students (1, 2). Existing studies have consistently shown that adverse childhood experiences (ACEs), including abuse, neglect, and family dysfunction, are associated with a higher risk of NSSI in adolescence and young adulthood (3–6). This evidence indicates that early adversity is an important risk source for later self-injurious behavior. However, previous studies have mainly focused on the direct association between ACEs or childhood trauma and NSSI, while the modifiable behavioral and psychological processes through which ACEs are linked to NSSI among college students remain insufficiently clarified (7–9).

Physical exercise (PE) is a modifiable health behavior with potential protective value for mental health. Prior research has suggested that regular exercise may relieve stress, improve mood, strengthen perceived self-control, and reduce emotional or behavioral problems (10, 11). At the same time, individuals with higher exposure to early adversity may be less likely to maintain regular exercise because ACEs can weaken self-efficacy, body-related confidence, and health behavior regulation (12, 13). Nevertheless, few studies have directly examined whether PE serves as a mediating pathway between ACEs and NSSI in college students.

Psychological resilience (PR) has also been identified as an important protective factor in the context of early adversity and NSSI (14). Previous work has shown that ACEs may undermine the development of resilience by damaging secure attachment, positive self-cognition, and emotion regulation resources, whereas higher PR is associated with lower levels of maladaptive coping behaviors, including NSSI (15–17). In addition, PE may help cultivate PR by providing mastery experiences, improving emotion regulation, and strengthening perceived control (18). These findings suggest a possible sequential pathway in which ACEs reduce PE, reduced PE weakens PR, and lower PR is associated with higher NSSI (19). However, this chain mediation pathway has not been sufficiently tested in college students.

Another limitation of previous research is that NSSI has often been examined using total scores, which may mask differences among specific self-injurious behaviors (20). Network analysis provides a symptom-level perspective by estimating conditional associations among individual behavioral items and identifying nodes with relatively high centrality (21–23). Although network methods have increasingly been applied in psychopathology, behavioral network analyses of NSSI among college students remain relatively limited (24). Therefore, symptom-level evidence may complement variable-level mediation analysis and provide a more detailed description of the internal structure of NSSI behaviors (25).

Based on the above literature, the present study aimed to examine the association between ACEs and NSSI among college students from two complementary perspectives (26). First, chain mediation analysis was used to test whether PE and PR explain the association between ACEs and the total NSSI score (27). Second, network analysis was used to describe the item-level structure of 12 NSSI behaviors (28). These two analyses were treated as complementary but parallel approaches: the mediation model addressed variable-level pathways, whereas the network model described symptom-item associations.

Accordingly, the present study proposed four hypotheses: Hypothesis 1: ACEs are positively associated with NSSI among college students. Hypothesis 2: Higher ACEs are associated with lower PE, which is further associated with higher NSSI. Hypothesis 3: Higher ACEs are associated with weaker PR, which is further associated with higher NSSI. Hypothesis 4: PE and PR play a chain-mediating role in the relationship between ACEs and NSSI, such that ACEs are associated with lower PE, which is further associated with weaker PR and higher NSSI.

## 2 Methods

### 2.1 Participants and procedures

From September to November 2025, students from first year to fourth year in eight universities across four provinces, namely Guizhou, Henan, Sichuan, and Shanxi, were selected as participants through convenience sampling. Baseline data were collected through an online questionnaire. The inclusion criteria were as follows: (1) full-time college student at one of the selected universities; (2) aged 18 years or older; (3) able to complete the questionnaire independently; and (4) provided informed consent. The exclusion criteria were refusal to participate, age below 18 years, incomplete responses, duplicate submissions, response times shorter than 300 seconds, and obvious response errors, including out-of-range demographic information, contradictory responses, or straight-line responses across the main scales. The survey platform required responses to all core items; therefore, no item-level missing data were present in the retained dataset. Duplicate responses were reduced by limiting each device or account to one submission and by checking repeated IP addresses and submission times during data screening. After informed consent was obtained, a total of 2,800 questionnaires were distributed. Following data screening, 2,626 valid questionnaires were ultimately obtained, with an effective response rate of 93.8%. A total of 174 questionnaires (6.2%) were excluded because they were incomplete, duplicate submissions, completed too quickly, or contained obvious response errors. Because many excluded questionnaires did not contain complete demographic and scale information, a reliable demographic comparison between the retained and excluded samples could not be conducted; this issue is acknowledged as a potential source of selection bias. All retained participants were adults; therefore, guardian consent was not required. The participants’ ages ranged from 18 to 24 years (M = 20.75, SD = 1.60). The demographic characteristics and sample distribution by province and university are shown in **Table 1**. Among them, 1,065 were male students (40.60%) and 1,561 were female students (59.40%); 769 were first-year students (29.30%), 806 were second-year students (30.70%), 615 were third-year students (23.40%), and 436 were fourth-year students (16.60%); 1,170 students were from urban areas (44.60%) and 1,456 were from rural areas (55.40%); 1,120 were only children (42.70%) and 1,506 were not an only children (57.30%).

**Table 1:** Descriptive statistics.

Variable	Category	Count	Column N %
Sex	Male	1,065	40.60%
	Female	1,561	59.40%
Grade	First year	769	29.30%
	Second year	806	30.70%
	Third year	615	23.40%
	Fourth year	436	16.60%
Family residence	Urban	1,170	44.60%
	Rural	1,456	55.40%
Not an only child	Yes	1,120	42.70%
	No	1,506	57.30%

This study was approved by the Ethics Committee of the School of Physical Education, Qiannan Normal University for Nationalities. Before completing the questionnaire, all participants read the informed consent statement and voluntarily agreed to participate. The survey was anonymous, and all data were used only for academic research.

### 2.2 Measures

#### 2.2.1 Adverse childhood experiences scale

The ACEs scale used in this study was used to assess severe and frequent adverse experiences in early life, with a particular focus on childhood abuse and neglect. In the present study, ACEs were assessed using a CTQ-based measure of childhood maltreatment, which captures the abuse and neglect components of ACEs. It was originally developed by Bernstein et al. (29), and later revised and introduced into China by Zhao Xingfu et al. (30). The scale includes five subscales: emotional abuse, physical abuse, sexual abuse, emotional neglect, and physical neglect. It uses a 5-point scoring method, corresponding to “never, occasionally, sometimes, often, and always.” Higher scores indicate higher levels of maltreatment-related adverse childhood experiences. This scale has been used in Chinese samples and has shown good reliability and validity. In the present study, the internal consistency reliability of the scale was good (Cronbach’s α = 0.940).

#### 2.2.2 Physical activity rating scale

The Physical Activity Rating Scale (PARS, often abbreviated as PARS or PARS-3) is a self-report questionnaire used to measure the amount of physical exercise performed by an individual within a certain period of time. It was originally developed by the Japanese scholar Hashimoto Kimio and revised by the Chinese scholar Liang Deqing in 1994 (31). This scale requires participants to conduct self-ratings on three dimensions of physical exercise, namely intensity, frequency, and duration of each exercise session, with each dimension rated on a 1–5 scale. The total physical exercise score is calculated as: intensity score × (duration score − 1) × frequency score, with a score range of 0–100. According to the total score, exercise level can be classified into small amount of exercise (≤19 points), moderate amount of exercise (20–42 points), and large amount of exercise (≥43 points). In the present study, the internal consistency reliability of this scale was good (Cronbach’s α = 0.845).

#### 2.2.3 Psychological resilience scale

The Connor-Davidson Resilience Scale (CD-RISC) is a tool used to measure an individual’s ability to positively adapt, recover, and maintain mental health when facing stress, adversity, and challenges. This scale was developed by Kathleen M. Connor and Jonathan R. T. Davidson in 2003 (32), and the Chinese version was revised by Xiao Nan and Zhang Jianxin in 2007 (33). Its validity has been verified among Chinese college students. The scale includes three dimensions, namely tenacity, strength, and optimism, covering assessment content such as self-confidence, adaptability, sense of control, and emotion regulation. It contains 25 items and uses a 1–5 scoring method (1 = very inconsistent, 5 = very consistent), with higher scores indicating a higher level of Psychological Resilience (PR). In the present study, the internal consistency reliability of this scale was good (Cronbach’s α = 0.886).

#### 2.2.4 Adolescent non-suicidal self-injury assessment questionnaire

The Adolescent Non-suicidal Self-injury Assessment Questionnaire (ANSAQ) is a tool specifically designed for Chinese adolescents to assess non-suicidal self-injury behavior and the underlying motivations. This questionnaire was developed by Wan Yuhui et al (34). It includes a 12-item behavioral questionnaire and uses a 0–4 scoring method (corresponding to “never, occasionally, sometimes, often, and always”), with higher total scores indicating more frequent or more severe self-injurious behavior. It has shown good reliability and validity among Chinese adolescent samples. In the present study, the Cronbach’s α coefficient of this questionnaire was 0.915.

### 2.3 Data analysis

In the present study, SPSS 27.0 and its PROCESS macro (Model 6) were used to test the chain mediating effect based on the 2,626 valid questionnaires collected. Network analysis was conducted in R 4.3.2 using the qgraph, bootnet, and networktools packages. The 12 NSSI items were treated as five-level ordered ordinal variables after correction to a 0–4 scoring range. Therefore, a polychoric correlation matrix was first estimated, and the network was then estimated using the EBIC graphical LASSO method (EBICglasso), with the EBIC tuning parameter γ set to 0.5. The edge weights represent regularized partial correlations, rather than raw zero-order correlations. Positive and negative edges indicate conditional associations between two NSSI items after controlling for all other items in the network. Expected influence was used as the main centrality index. Edge-weight accuracy and centrality stability were evaluated using nonparametric bootstrap confidence intervals and case-dropping bootstrap procedures with 1,000 bootstrap samples. Edge-weight difference tests, centrality difference tests, and node predictability analyses were not conducted. Before mediation and network analyses, the distributions of PE and NSSI were examined. Because the PE score was positively skewed, a sensitivity analysis using log-transformed PE [ln(PE + 1)] was additionally conducted for the mediation model.

## 3 Results

### 3.1 Common method bias test

Procedural and statistical controls were used to reduce and examine common method bias. Procedurally, the questionnaire was completed anonymously, there were no right or wrong answers, and participants were informed that the data would be used only for academic research. Statistically, Harman’s single-factor test showed that a total of 10 independent common factors with eigenvalues greater than 1 were identified, and the first factor explained 20.78% of the total variance, below the 40% threshold. In addition, a full collinearity check was conducted as a supplementary diagnostic. The VIF values for the main variables ranged from 1.212 to 1.226, all below the commonly used threshold of 3.3. These results suggested that serious common method bias was unlikely, although it could not be completely ruled out because all main variables were measured using self-report questionnaires at the same time point.

### 3.2 Descriptive statistics and correlation analysis

Correlation analyses were conducted on the mean scores of ACEs, PR, NSSI, and PE averaged item scores of ACEs, PR, and NSSI, together with the PE total score. The results showed that ACEs were positively correlated with NSSI and negatively correlated with PR and PE; PR was negatively correlated with NSSI and positively correlated with PE; NSSI was also negatively correlated with PE (see **Table 2**).

**Table 2:** Means, standard deviations, and correlation coefficients of all variables.

Variable	*M ± SD*	ACEs	PR	NSSI	PE
ACEs	3.08 ± 0.94	1			
PR	3.07 ± 0.73	-0.314***	1		
NSSI	1.09 ± 0.99	0.329***	-0.271***	1	
PE	29.36 ± 31.26	-0.266***	0.340***	-0.322***	1

***p < 0.001. The same notation applies below. ACEs, adverse childhood experiences; PR, psychological resilience; NSSI, non-suicidal self-injury; PE, physical exercise.

### 3.3 Mediation effect test

In the present study, ACEs were treated as the independent variable (X), PE and PR were treated as the mediating variables (M1 and M2), and NSSI was treated as the dependent variable (Y). The theory-driven statistical indirect associations among the variables were examined using the PROCESS V4.1 macro (Model 6) in SPSS 27.0. In the model, sex, age, grade, family residence, and only-child status were controlled as covariates. The results of the hierarchical regression analysis are shown in **Table 3**. The coefficients reported in **Table 3** are unstandardized regression coefficients (B). Specifically, ACEs were significantly and negatively associated with PE (B = -0.858, p < 0.001) and PR (B = -0.188, p < 0.001), and significantly and positively associated with NSSI (B = 0.244, p < 0.001). PE was significantly and positively associated with PR (B = 0.007, p < 0.001), and significantly and negatively associated with NSSI (B = -0.007, p < 0.001). In addition, PR was also significantly and negatively associated with NSSI (B = -0.162, p < 0.001). The bootstrap test further showed (see **Table 4**) that the 95% confidence intervals of the three indirect pathways, namely “ACEs → PE → NSSI,” “ACEs → PR → NSSI,” and “ACEs → PE → PR → NSSI,” did not include zero, indicating that the statistical indirect effects were significant. The total effect was 0.345, the direct effect was 0.245, and the total indirect effect was 0.100. The three specific indirect effects summed to 0.100 (0.061 + 0.030 + 0.009), which was equal to the difference between the total and direct effects (0.345 - 0.245 = 0.100).

**Table 3:** Regression results for the chain mediation model.

Outcome variable	Predictor variable	*R*	*R^2^*	*F*	B	*t*
PE	ACEs	0.268	0.072	33.825	-0.858	-14.151
	Sex				-0.049	-0.040
	AGE				-0.092	-0.261
	Grade				0.269	0.480
	Family residence				0.504	0.425
	Not an only child				1.767	1.479
PR	ACEs	0.416	0.173	78.076	-0.188	-13.046
	PE				0.007	14.994
	Sex				-0.021	-0.798
	AGE				-0.007	-0.866
	Grade				-0.039	-3.101
	Family residence				0.014	0.516
	Not an only child				-0.018	-0.682
NSSI	ACEs	0.433	0.187	75.379	0.244	12.316
	PE				-0.007	-11.457
	PR				-0.162	-6.195
	Sex				-0.070	-1.961
	AGE				-0.006	-0.523
	Grade				0.063	3.813
	Family residence				-0.022	-0.635
	Not an only child				-0.048	-1.354

B represents the unstandardized regression coefficient. Sex, age, grade, family residence, and only-child status were included as covariates.

**Table 4:** Total, direct, and indirect effects.

Effect	Effect size	Bootstrap standard error	LLCI	ULCI
Total	0.345	0.019	0.307	0.383
Direct	0.245	0.020	0.205	0.283
Total indirect effect	0.100	0.009	0.083	0.118
ACEs→PE→NSSI	0.061	0.007	0.048	0.074
ACEs→PR→NSSI	0.030	0.005	0.020	0.041
ACEs→PE→PR→NSSI	0.009	0.002	0.006	0.013

Effect size represents unstandardized total, direct, total indirect, and specific indirect effects. Bootstrap confidence intervals were based on 5,000 resamples. LLCI/ULCI, lower/upper limits of the 95% confidence interval.

### 3.4 Network analysis

**Figure 1:**
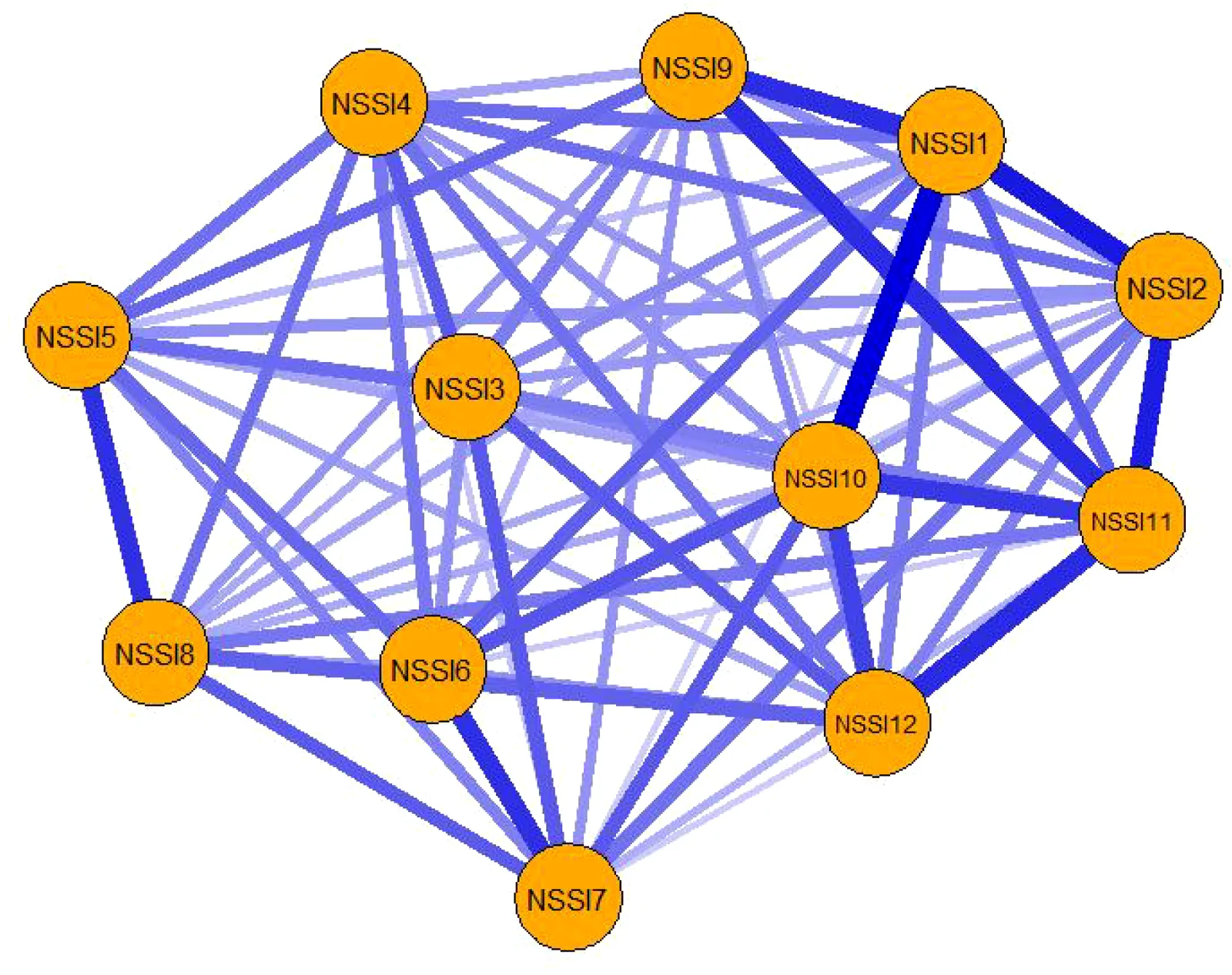
Cross-sectional network structure. The network was estimated using EBICglasso based on a polychoric correlation matrix. Nodes represent NSSI behavioral items, and edges represent regularized partial correlations between two items after controlling for all other items in the network. Blue edges indicate positive associations. Thicker and darker edges indicate larger edge weights. The network is undirected; therefore, the edges indicate conditional associations rather than causal or temporal directions.

Based on the results of the cross-sectional network analysis (see **Figure 1**), the structural characteristics of the NSSI symptom network among college students were examined. Among all regularized network edges, the two relatively strongest connections were found between “deliberately pinching oneself” (NSSI1) and “deliberately burning or scalding oneself” (NSSI10) (edge weight = 0.159), and between “deliberately pinching oneself” (NSSI1) and “deliberately scratching oneself” (NSSI2) (edge weight = 0.139). These edge weights were modest and should be interpreted as the relatively strongest conditional associations within this network, rather than as evidence of a causal sequence. Further, according to the expected influence centrality index, NSSI1 had the highest expected influence value in the network (EI = 1.764), suggesting that this node was relatively central in the NSSI symptom network. Taken together, these findings indicate that pinching behavior is a noteworthy symptom for further attention, but the cross-sectional network cannot demonstrate that pinching is a gateway symptom or that intervening on pinching would reduce other NSSI behaviors.

In the centrality analysis, the present study selected Expected Influence (EI) as the indicator for measuring the importance of nodes in the network, mainly based on two considerations. First, in a mixed-polarity psychological network containing both positive and negative edges, expected influence not only takes into account the strength of node connections, but also considers the direction of the connections (positive or negative). Therefore, compared with traditional indicators such as strength centrality, it has better stability and interpretability. Second, the stability test results (CS > 0.5) indicated that expected influence had acceptable measurement stability in the network of the present study and could relatively reliably reflect the structural characteristics of the nodes.

**Figure 2:**
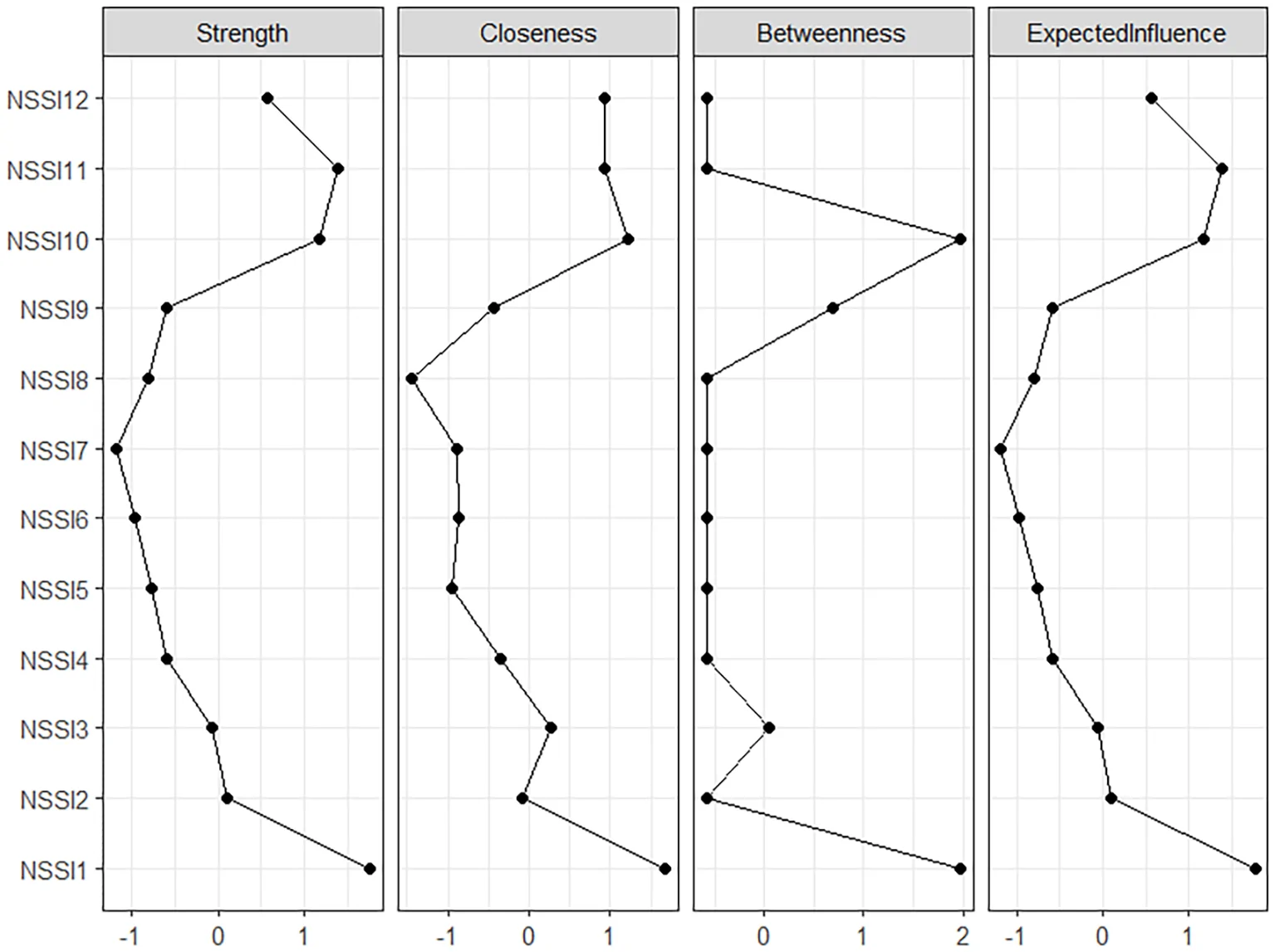
Distribution of expected influence centrality values of nodes in the overall network.

According to the results of the centrality analysis (see **Figure 2**), the standardized expected influence values of the nodes showed differences. Among them, “deliberately pinching oneself” (NSSI1) had the highest standardized expected influence value (EI = 1.764), indicating that this node had the highest relative centrality in the current network. In contrast, some nodes, such as “deliberately stabbing or poking oneself” (NSSI6) and “deliberately cutting oneself” (NSSI7), had negative standardized expected influence values (EI = -0.974 and -1.182, respectively). These negative values indicate that the corresponding nodes were below the network average in standardized expected influence; they do not mean that these symptoms were inhibited or regulated by other symptoms. In summary, NSSI1 was the most central node in this cross-sectional network, but this finding should be interpreted as a descriptive network result rather than evidence of a causal or intervention target.

To examine the stability of the cross-sectional network, the present study conducted a stability analysis using the bootstrap method. The 95% bootstrap confidence intervals of the edge weights were generally narrow (see **Supplementary Figure S1**), indicating that the estimates of connection strength in the network were relatively stable. Furthermore, the stability coefficients (CScoefficient) were calculated using the case-dropping bootstrap procedure. The results showed that the stability coefficient for edges was 0.439, the stability coefficients for eigenvector centrality and expected influence were both 0.75 (both representing the highest level within the tested range), and the stability coefficient for strength centrality was 0.672. All of the above stability coefficients were greater than the acceptable criterion of 0.25, indicating that the network had moderate to good stability in key features such as connection strength and expected influence (for related results, see **Supplementary Figure S2**).

## 4 Discussion

### 4.1 The association between ACEs and NSSI among college students

The results of the present study showed that ACEs were significantly associated with NSSI among college students, and Hypothesis 1 was supported (35). This finding is consistent with many previous studies, all of which indicate that exposure to early adversity is an important correlate of self-injurious behavior in adolescence and young adulthood (36). At the theoretical level, according to the developmental cascade model of psychological trauma, the early chronic stress represented by ACEs may interfere with the development of individuals’ emotion regulation systems and self-cognition, leading to long-term emotional dysregulation and self-devaluation tendencies, thereby increasing the likelihood that individuals adopt NSSI as a means of emotional release or self-punishment when facing subsequent stress (37–40). In the Chinese sociocultural context, this association may be further strengthened by the following factors. First, the parenting style of “high demands and low emotional responsiveness,” which may exist in traditional family values, may intensify the inhibitory effect of ACEs on individuals’ emotional expression and help-seeking behaviors (41, 42). Second, in a highly competitive academic and employment environment, college students who have experienced ACEs may lack sufficient social support and psychological resources to cope with developmental challenges, making them more likely to fall into a behavioral pattern of using self-injury to deal with stress (43–45). Therefore, the findings of the present study not only reconfirm the potential association ACEs on NSSI at the empirical level, but also suggest that the prevention of early trauma and psychological intervention are of great importance among Chinese college students.

### 4.2 The mediating role of PE between ACEs and NSSI

The results of the chain mediation analysis showed that PE played a significant statistical indirect role between ACEs and NSSI among college students, and Hypothesis 2 was supported (46). This finding indicates that higher ACEs were associated with lower PE, and lower PE was further associated with higher NSSI (47–49). However, this result should be interpreted as a theory-driven cross-sectional indirect association, not as evidence that ACEs causally reduce PE or that lower PE causally increases NSSI.

On the one hand, the negative effect of ACEs on physical exercise can be understood from the social-ecological model of health behavior (50). This theory emphasizes that individual health behavior is influenced by multiple systems, and early traumatic environments may damage the psychological and social foundations required for individuals to develop regular exercise habits (51–54). Specifically, ACEs may reduce exercise behavior by weakening bodily autonomy, lowering exercise self-efficacy, and disrupting individuals’ connections with supportive exercise environments, such as family encouragement and peer participation (55, 56). In the context of Chinese college students, this pathway may be particularly evident. Individuals who have experienced ACEs may lack family guidance or resources for developing exercise habits during their growth process (57, 58). After entering university, the pressure of academic competition and the requirement for relatively independent life management may make it more difficult for them to actively maintain regular exercise in the absence of an early foundation (59–61).

On the other hand, the inhibitory effect of physical exercise on NSSI can be theoretically supported by the stress-buffering hypothesis in sport and exercise psychology (62, 63). This hypothesis suggests that regular physical activity can enhance individuals’ physiological and psychological resistance to stress (64, 65). From a physiological perspective, exercise can regulate the functioning of the hypothalamic-pituitary-adrenal axis and reduce the basal level and reactivity of stress hormones such as cortisol, thereby alleviating persistent emotional tension (66, 67). From a psychosocial perspective, exercise provides individuals with a sense of control, positive emotional experiences, and alternative social opportunities, all of which may reduce reliance on maladaptive emotion regulation strategies such as NSSI (68, 69). A large number of empirical studies have also shown that adolescents and young adults with higher levels of physical activity participation tend to have relatively lower rates of emotional dysregulation and self-injurious behavior (70, 71).

In summary, the present study revealed the mediating role of PE in the association between ACEs and NSSI. This not only provides a behavioral-level explanation for the mechanism through which ACEs affect NSSI, but also suggests that promoting and facilitating physical exercise may be a feasible intervention pathway for interrupting the subsequent psychological risks of early adversity and improving the mental health of college students (11, 72).

### 4.3 The mediating role of PR between ACEs and NSSI

The results of the chain mediation analysis further showed that PR played a significant statistical indirect role between ACEs and NSSI among college students, and Hypothesis 3 was supported (73, 74). This suggests that higher ACEs were associated with weaker PR, which was further associated with higher NSSI (75). Given the cross-sectional design, this pattern should be understood as a statistical association rather than a demonstrated causal process.

On the one hand, the erosive effect of ACEs on PR can be theoretically explained by the resource depletion model of resilience (76). This model holds that the formation and development of resilience depend on the continuous accumulation of key psychological resources, such as secure attachment, positive cognitive schemas, and effective coping skills (77, 78). As a chronic developmental stressor, ACEs may continuously consume or hinder the acquisition of these resources (79). For example, they may damage individuals’ basic sense of security, foster negative beliefs about the self and the world, and restrict the learning of adaptive coping strategies, thereby leading to insufficient resilience reserves when individuals later face stress (80). In the context of Chinese society and culture, this process may be intensified by certain factors. Under narratives that emphasize academic achievement and “being strong,” the emotional pain of individuals with ACEs may be less likely to be identified and accepted in a timely manner, and family and social support systems may fail to effectively intervene to compensate for the loss of early resources, thus hindering the development of PR (81).

On the other hand, the buffering effect of PR on NSSI can be understood on the basis of the stress-buffering theory of resilience. This theory suggests that high resilience helps individuals evaluate and cope with stressful events in a more positive and flexible way, reduces the intensity and duration of negative emotions, and enhances the tendency to use adaptive strategies such as problem solving and seeking support. This reduces the likelihood that individuals will resort to NSSI, a harmful and functionally limited form of emotion regulation. Specifically, individuals with higher levels of PR may have better abilities in emotional awareness and regulation, higher self-efficacy, and broader social support networks. These protective factors jointly reduce the impulse and need to engage in self-injurious behavior during emotional crises. A large body of empirical research has also consistently found that PR is significantly negatively associated with emotional and behavioral problems, including NSSI, among adolescents and young adults.

In summary, the present study confirmed the key mediating role of PR in the association between ACEs and NSSI. This finding not only deepens the understanding of the psychological mechanism underlying the long-term effects of early adversity and highlights the importance of PR as a core protective factor, but also provides a clear direction for mental health interventions targeting college students. That is, by focusing on the cultivation and enhancement of PR, it may be possible to effectively interrupt the negative chain associated with ACEs and strengthen individuals’ ability to resist the risk of self-injury.

### 4.4 The chain mediating role of PE and PR

Through the chain mediation model, the present study found that PE and PR constituted a significant sequential mediating pathway between ACEs and NSSI among college students, and Hypothesis 4 was supported (82). This result reveals that ACEs may first reduce individuals’ physical exercise behavior, then weaken the development of their PR, and ultimately partially lead to an increased risk of NSSI.

The existence of this chain pathway can be theoretically explained from the biopsychosocial integrative perspective of health psychology. First, as an observable and adjustable external behavior, PE is an important entry point for shaping internal psychological resources (83). According to self-efficacy theory, regular participation in physical exercise can provide individuals with continuous “mastery experiences (84).” This sense of successful control over the body may generalize into broader beliefs in personal efficacy, which is precisely a core cognitive component of PR (85). At the same time, from a physiological perspective, physical exercise can promote neuroplasticity related to emotion regulation and cognitive functioning, such as enhancing prefrontal functioning and increasing the level of brain-derived neurotrophic factor, which lays a biological foundation for the “flexibility of emotion regulation” and the “cognitive reappraisal of stress” required for PR (86). Therefore, a reduction in PE means that individuals lose an active pathway for continuously cultivating key components of PR, such as self-efficacy and neurophysiological foundations (52).

When this mechanism is placed in the developmental context of Chinese college students, its practical significance becomes particularly prominent. During the university stage, individuals transition from the family environment to relatively autonomous collective life (87). If students who have experienced ACEs fail to establish regular physical exercise habits, they may miss a key opportunity to actively build psychological resources and cope with the pressure of a new environment in the early stage of independent living (88). Especially in an atmosphere oriented toward academic achievement, the marginalization of physical exercise may further amplify the “exercise gap” in their PR, making them more likely to fall into a state of resource depletion when facing common challenges such as academic competition and interpersonal stress, and increasing the risk of using NSSI as a coping strategy (89).

In summary, the chain mediation finding of the present study clarifies a coherent and intervenable “behavior–psychology” pathway from early adversity to maladaptive behavior (90). This suggests that preventive interventions for college students should not only focus on the direct enhancement of PR, but may also take the promotion of regular PE as a prioritized and operable behavioral lever (91). Through behavioral change, PR resources may be systematically nourished, thereby more effectively interrupting the risk transmission process from ACEs to NSSI (92).

### 4.5 Network analysis: identifying noteworthy symptoms in the NSSI symptom network

Through network analysis, the present study examined the internal structure of the NSSI behavioral system among college students at the symptom level and found that “deliberately pinching oneself” had the highest expected influence in the symptom network (93). This node was also involved in the two relatively strongest, although modest, edges in the network, namely its connections with “deliberately burning or scalding oneself” and “deliberately scratching oneself” (94). This finding suggests that pinching behavior is a noteworthy symptom within the NSSI network. However, because the present network was cross-sectional, it cannot demonstrate that pinching is a “gateway symptom,” a “core transmitter,” or a causal driver of other self-injurious behaviors. Therefore, the network results should be interpreted as exploratory structural evidence that may inform future longitudinal and intervention research.

At the macro level, regular physical exercise that can enhance the sense of control and pleasure may be systematically promoted through university physical education courses, club activities, and campus environmental modification, so that it serves as a general strategy for cultivating PR and strengthening students’ ability to cope with psychological risks (95, 96). At the symptom level, the relatively high centrality of pinching suggests that this behavior deserves attention during screening, assessment, and follow-up among students with self-injury risk. However, the present study does not provide direct evidence that targeting pinching alone would reduce other NSSI behaviors. Future longitudinal and intervention studies are needed to determine whether changes in this behavior lead to changes in the broader NSSI symptom network.

## 5 Theoretical and practical implications

The theoretical significance of the present study lies in its use of two complementary analytical perspectives. The chain mediation model supported a sequential protective pathway in which ACEs were associated with NSSI through lower PE and weaker PR. The network analysis, conducted separately at the item level, further described the internal structure of NSSI behaviors and identified pinching as the node with the highest expected influence in this cross-sectional network. These findings should not be interpreted as a single integrated statistical model linking ACEs, PE, PR, and pinching. Rather, they provide parallel evidence from the total-score level and the symptom-item level, which may help future studies further examine how general risk pathways relate to specific NSSI behaviors.

The practical significance of the present study is mainly reflected in its implications for prevention and assessment. First, the study identified PE as a feasible and easily promotable behavioral factor associated with the ACEs-NSSI pathway, which provides empirical support for colleges and universities to integrate mental health promotion into physical education, club activities, and campus culture construction. Second, network analysis highlighted “pinching” as a noteworthy but not causally confirmed symptom in the NSSI network. This finding may help school mental health practitioners pay closer attention to specific self-injurious behaviors during screening and follow-up. However, the present analyses do not show that ACEs, PE, or PR are specifically associated with pinching, nor do they demonstrate that intervening on pinching alone would reduce other NSSI behaviors.

## 6 Limitations and future directions

The present study has several limitations. First, its cross-sectional design does not allow for strict causal inference among variables. Future studies may adopt longitudinal tracking or experimental intervention designs to verify the causal pathways through which PE and PR operate between ACEs and NSSI. In addition, the study relied on self-report scales for measurement, and the sample was drawn from universities in specific provinces, which may limit the generalizability of the findings. Future research may improve validity by combining objective indicators, such as data from wearable activity trackers, and by sampling across broader geographical and cultural contexts. At the same time, the chain mediation model and the NSSI item network were parallel analyses rather than a single integrated statistical model. Therefore, the present findings cannot demonstrate that ACEs, PE, or PR are specifically associated with pinching. Future studies may include ACEs, PE, PR, and NSSI behaviors in a unified network or longitudinal model to further examine these associations. Finally, because the network analysis was cross-sectional and node predictability was not estimated, the centrality results should be interpreted as descriptive indicators of the current network rather than as direct evidence for intervention effects. In addition, although procedural controls and supplementary statistical checks were used, the cross-sectional self-report design means that common method bias cannot be fully excluded.

## 7 Conclusion

The present study examined the association between ACEs and NSSI among college students from two complementary perspectives. The chain mediation analysis showed a “behavior-psychology” pathway from early adversity to self-injurious behavior: ACEs not only directly increased the risk of NSSI, but also indirectly increased this risk by reducing individuals’ participation in PE, which in turn weakened their PR. The network analysis further showed that “deliberately pinching oneself” had the highest expected influence in the NSSI behavioral network and was involved in the relatively strongest edges. This finding should be interpreted as a descriptive cross-sectional network result rather than evidence of a gateway symptom or confirmed intervention target. Overall, the findings provide parallel evidence at the variable and symptom levels for understanding NSSI among college students.

## Data Availability

The original contributions presented in the study are included in the article/**Supplementary Material**. Further inquiries can be directed to the corresponding authors.
