# Supplementary material for: Physical exercise and psychological resilience in the link between adverse childhood experiences and non-suicidal self-injury: chain mediation and network analysis in college students
Source: Front Psychiatry. 2026 Aug 12;17:1889977. doi: 10.3389/fpsyt.2026.1889977 (PMC13506935; doi:10.3389/fpsyt.2026.1889977)
Supplement: Supplementary file 1 [file SupplementaryFile1.docx]

Supplementary Material

# 1 Supplementary Figures and Tables

## 1.1 Supplementary Tables

Table S-1 Distributional Characteristics and Endorsement of Non-Suicidal Self-Injury

Panel A. Overall NSSI mean score and positive status

| Statistic | Value |
| --- | --- |
| Score used in analyses | Mean of the 12 corrected items |
| Score range | 0-4 |
| Mean ± SD | 1.086 ± 0.988 |
| Median | 1.167 |
| IQR | 1.50 |
| Skewness | 0.037 |
| Excess kurtosis | -1.118 |
| NSSI-positive, n (%) | 2432 (92.6%) |

Panel B. Item-level distribution and endorsement rates

| Variable | Item | Label | Range | M | SD | Median | IQR | Skewness | Excess kurtosis | Zero score, n (%) | Endorsement, n (%) |
| --- | --- | --- | --- | --- | --- | --- | --- | --- | --- | --- | --- |
| NSSI behavior | Intentionally pinching oneself to cause injury | NSSI1 | 0-4 | 1.040 | 1.426 | 1.00 | 2.00 | -0.066 | -1.343 | 523 (19.9%) | 1581 (60.2%) |
|  | Intentionally scratching oneself to cause injury | NSSI2 | 0-4 | 1.045 | 1.395 | 1.00 | 2.00 | -0.088 | -1.274 | 493 (18.8%) | 1630 (62.1%) |
|  | Intentionally banging one's head against hard objects (e.g., walls, trees, etc.) | NSSI3 | 0-4 | 1.018 | 1.381 | 1.00 | 2.00 | -0.050 | -1.250 | 512 (19.5%) | 1617 (61.6%) |
|  | Intentionally hitting walls, tables, windows, floors, or other hard objects with one's fist | NSSI4 | 0-4 | 1.111 | 1.358 | 1.00 | 2.00 | -0.158 | -1.195 | 485 (18.5%) | 1702 (64.8%) |
|  | Intentionally injuring oneself with fists, slaps, or hard objects | NSSI5 | 0-4 | 1.081 | 1.362 | 1.00 | 2.00 | -0.132 | -1.219 | 503 (19.2%) | 1669 (63.6%) |
|  | Intentionally stabbing or puncturing oneself (e.g., with needles, staples, pen tips, etc.) | NSSI6 | 0-4 | 1.113 | 1.350 | 1.00 | 2.00 | -0.160 | -1.202 | 518 (19.7%) | 1685 (64.2%) |
|  | Intentionally cutting oneself (e.g., with blades, glass, etc.) | NSSI7 | 0-4 | 1.071 | 1.367 | 1.00 | 2.00 | -0.143 | -1.212 | 461 (17.6%) | 1685 (64.2%) |
|  | Intentionally biting oneself | NSSI8 | 0-4 | 1.134 | 1.346 | 1.00 | 2.00 | -0.172 | -1.169 | 488 (18.6%) | 1723 (65.6%) |
|  | Intentionally pulling out one's own hair | NSSI9 | 0-4 | 1.160 | 1.365 | 1.00 | 2.00 | -0.232 | -1.181 | 439 (16.7%) | 1746 (66.5%) |
|  | Intentionally burning or scalding oneself (e.g., with cigarette butts, boiling water, lighters, or matches, etc.) | NSSI10 | 0-4 | 1.087 | 1.368 | 1.00 | 2.00 | -0.135 | -1.212 | 477 (18.2%) | 1687 (64.2%) |
|  | Intentionally rubbing skin with objects to cause bleeding or bruising | NSSI11 | 0-4 | 1.070 | 1.379 | 1.00 | 2.00 | -0.106 | -1.236 | 487 (18.5%) | 1664 (63.4%) |
|  | Intentionally carving words or symbols into skin (excluding tattoos) | NSSI12 | 0-4 | 1.100 | 1.386 | 1.00 | 2.00 | -0.121 | -1.252 | 500 (19.0%) | 1667 (63.5%) |

**Table S-2 Cross-Sectional Network Edge Weights (Regularized Partial Correlations; Top 30)**

| Rank | Node |  | Node | ***Edge weight (regularized partial correlation)*** |
| --- | --- | --- | --- | --- |
| 1 | NSSI1 | - | NSSI10 | 0.159 |
| 2 | NSSI1 | - | NSSI2 | 0.139 |
| 3 | NSSI2 | - | NSSI11 | 0.137 |
| 4 | NSSI11 | - | NSSI12 | 0.129 |
| 5 | NSSI9 | - | NSSI11 | 0.129 |
| 6 | NSSI1 | - | NSSI9 | 0.127 |
| 7 | NSSI6 | - | NSSI7 | 0.126 |
| 8 | NSSI5 | - | NSSI8 | 0.126 |
| 9 | NSSI10 | - | NSSI11 | 0.120 |
| 10 | NSSI10 | - | NSSI12 | 0.116 |
| 11 | NSSI6 | - | NSSI10 | 0.105 |
| 12 | NSSI7 | - | NSSI10 | 0.103 |
| 13 | NSSI3 | - | NSSI7 | 0.102 |
| 14 | NSSI1 | - | NSSI11 | 0.102 |
| 15 | NSSI7 | - | NSSI8 | 0.102 |
| 16 | NSSI3 | - | NSSI12 | 0.102 |
| 17 | NSSI3 | - | NSSI4 | 0.101 |
| 18 | NSSI1 | - | NSSI6 | 0.098 |
| 19 | NSSI8 | - | NSSI12 | 0.098 |
| 20 | NSSI5 | - | NSSI9 | 0.094 |
| 21 | NSSI5 | - | NSSI6 | 0.094 |
| 22 | NSSI3 | - | NSSI5 | 0.092 |
| 23 | NSSI1 | - | NSSI4 | 0.091 |
| 24 | NSSI4 | - | NSSI8 | 0.090 |
| 25 | NSSI2 | - | NSSI4 | 0.090 |
| 26 | NSSI4 | - | NSSI5 | 0.089 |
| 27 | NSSI4 | - | NSSI12 | 0.088 |
| 28 | NSSI2 | - | NSSI7 | 0.088 |
| 29 | NSSI8 | - | NSSI11 | 0.085 |
| 30 | NSSI4 | - | NSSI6 | 0.085 |

Note. Edge weights are regularized partial correlations estimated using EBICglasso based on the polychoric correlation matrix; they are not raw zero-order correlation coefficients.

**Table S-3** Standardized Expected Influence Scores for Each Node in the Network

| Rank | Node | Standardized expected influence |
| --- | --- | --- |
| 1 | NSSI1 | 1.764 |
| 2 | NSSI2 | 0.099 |
| 3 | NSSI3 | -0.069 |
| 4 | NSSI4 | -0.591 |
| 5 | NSSI5 | -0.767 |
| 6 | NSSI6 | -0.974 |
| 7 | NSSI7 | -1.182 |
| 8 | NSSI8 | -0.803 |
| 9 | NSSI9 | -0.591 |
| 10 | NSSI10 | 1.170 |
| 11 | NSSI11 | 1.382 |
| 12 | NSSI12 | 0.562 |

Table S-4 Eligibility, Missing-Data Handling, and Data-Quality Screening

| Item | Description |
| --- | --- |
| Initial questionnaires distributed | 2,800 |
| Valid questionnaires retained | 2,626 (93.8%) |
| Age eligibility | Only students aged 18 years or older were allowed to complete and submit the questionnaire. |
| Inclusion criteria | Full-time college student at a selected university; age ≥18 years; able to complete the questionnaire independently; informed consent provided. |
| Exclusion criteria | No informed consent, age <18 years, incomplete questionnaire, duplicate submission, response time shorter than 300 seconds, out-of-range demographic information, contradictory responses, or straight-line responses across the main scales. |
| Missing data | The survey platform required responses to all core items. Incomplete questionnaires were excluded before analysis; therefore, no item-level missing data were present in the retained dataset. |
| Duplicate-response control | Each device or account was limited to one submission, and repeated IP addresses and submission times were checked during data screening. |
| Ethical consent | All retained participants were adults; therefore, guardian consent was not required. All participants provided informed consent online. |
| Family residence | Family residence was self-reported and coded as urban or rural. The previous wording 'family socioeconomic status' was corrected to 'family residence'. |

## 1.2 Supplementary Figures

**Figure S-1** Bootstrap confidence intervals for network edge weights

Figure S-2 Case-dropping bootstrap results for centrality stability
